# Maintenance of Intestinal Th17 Cells and Reduced Microbial Translocation in SIV-infected Rhesus Macaques Treated with Interleukin (IL)-21

**DOI:** 10.1371/journal.ppat.1003471

**Published:** 2013-07-04

**Authors:** Suresh Pallikkuth, Luca Micci, Zachary S. Ende, Robin I. Iriele, Barbara Cervasi, Benton Lawson, Colleen S. McGary, Kenneth A. Rogers, James G. Else, Guido Silvestri, Kirk Easley, Jacob D. Estes, Francois Villinger, Savita Pahwa, Mirko Paiardini

**Affiliations:** 1 University of Miami Miller School of Medicine, Miami, Florida, United States of America; 2 Division of Microbiology and Immunology, Yerkes National Primate Research Center, Emory University, Atlanta, Georgia, United States of America; 3 Department of Pathology and Laboratory Medicine, Emory University, Atlanta, Georgia, United States of America; 4 Department of Biostatistics and Bioinformatics, Rollins School of Public Health, Atlanta, Georgia, United States of America; 5 AIDS and Cancer Virus Program, Frederick National Laboratory for Cancer Research, SAIC-Frederick, Frederick, Maryland, United States of America; National Institute of Allergy and Infectious Diseases, National Institutes of Health, United States of America

## Abstract

In pathogenic HIV and SIV infections of humans and rhesus macaques (RMs), preferential depletion of CD4^+^ Th17 cells correlates with mucosal immune dysfunction and disease progression. Interleukin (IL)-21 promotes differentiation of Th17 cells, long-term maintenance of functional CD8^+^ T cells, and differentiation of memory B cells and antibody-secreting plasma cells. We hypothesized that administration of IL-21 will improve mucosal function in the context of pathogenic HIV/SIV infections. To test this hypothesis, we infected 12 RMs with SIV_mac239_ and at day 14 post-infection treated six of them with rhesus rIL-21-IgFc. IL-21-treatment was safe and did not increase plasma viral load or systemic immune activation. Compared to untreated animals, IL-21-treated RMs showed (i) higher expression of perforin and granzyme B in total and SIV-specific CD8^+^ T cells and (ii) higher levels of intestinal Th17 cells. Remarkably, increased levels of Th17 cells were associated with reduced levels of intestinal T cell proliferation, microbial translocation and systemic activation/inflammation in the chronic infection. In conclusion, IL-21-treatment in SIV-infected RMs improved mucosal immune function through enhanced preservation of Th17 cells. Further preclinical studies of IL-21 may be warranted to test its potential use during chronic infection in conjunction with antiretroviral therapy.

## Author Summary

In the gastrointestinal tract, preferential depletion of CD4^+^ Th17 cells occurs during the early stage of pathogenic HIV/SIV infections and correlates with loss of mucosal integrity, microbial translocation, immune activation and disease progression. As such, therapeutic intervention aimed at preserving intestinal Th17 cells may be of critical importance. IL-21 plays an important role in promoting the differentiation and survival of Th17 cells, as well as in stimulating CD8^+^ T cell cytolytic function. Here, we treated SIV-infected rhesus macaques with IL-21-IgFc in the early stage of infection. Consistent with the main functions of IL-21, we found that IL-21 treated animals had higher expression of perforin and granzyme B in total and SIV-specific CD8^+^ T cells and higher frequencies of intestinal Th17 cells as compared to untreated controls. Remarkably, the increased proportions of Th17 cells during early infection was associated with significantly lower levels of intestinal T cell proliferation, microbial translocation and systemic activation/inflammation during chronic infection. Thus, our results suggest that IL-21 treatment in SIV-infected RMs is effective in preserving intestinal Th17 cells and results in an improvement of mucosal immune function. Further preclinical studies may be warranted to test IL-21 during chronic infection and in conjunction with antiretroviral therapy.

## Introduction

Pathogenic Human Immunodeficiency Virus (HIV) and Simian Immunodeficiency Virus (SIV) infections in humans and RMs, respectively, are characterized by the establishment of a state of persistent and aberrant activation of the immune system [1], [2]. Two key findings highlight the importance of immune activation to disease progression in HIV/SIV infections. First, the level of chronic immune activation is a strong independent predictor of disease progression in the natural history of HIV infection, and associates with impaired immune reconstitution in HIV-infected individuals on antiretroviral therapy (ART) [3], [4], [5]. Second, the absence of chronic immune activation is a central feature of nonpathogenic SIV infections in natural hosts such as sooty mangabeys (SMs), African green monkeys (AGMs) and Mandrills [6], [7]. While the causes of immune activation during chronic HIV/SIV infections are complex and not fully defined, several studies indicated that mucosal immune dysfunction and associated loss of mucosal barrier integrity are key contributors to this process. In particular, the HIV/SIV-associated mucosal immune dysfunction appears to favor the translocation of microbial products from the intestinal lumen into the systemic circulation, where these products activate various innate immune pathways and exert a sustained pro-inflammatory effect [8], [9], [10], [11]. Chronic immune activation related to loss of mucosal barrier integrity and microbial translocation has been implicated in other pathological conditions including graft versus host disease, inflammatory bowel disease, and pancreatitis (reviewed in [12]). Enterocyte apoptosis [9], [13], massive loss of mucosal CD4^+^ T cells [14], [15], [16] and/or preferential loss of intestinal Th17 cells [17] have all been proposed as factors contributing to the breakdown of epithelial integrity during chronic HIV/SIV infections.

Several lines of evidence indicate that Th17 cells, and their signature cytokines IL-17 and IL-22, play a key role in the maintenance of structural and immunological integrity of mucosal sites [18], [19]. Effects of IL-17 and IL-22 include (i) stimulation of epithelial cells to express cytokines, chemokines and metalloproteinases involved in the recruitment, activation and migration of neutrophils to areas of bacterial infection (reviewed in [20]); (ii) production of antimicrobial molecules, such as defensins, by various cell types [21], [22]; and (iii) preservation of the integrity of the epithelial barrier by stimulating the proliferation of enterocytes and the transcription of tight-junction proteins, such as claudins [10], [23]. Consistent with these biological activities are the findings that Th17 cells confer protection against various extracellular pathogens, and that Th17 cell-mediated proinflammatory activity, when not properly regulated, may result in tissue damage and development of autoimmunity [24], [25]. Intestinal Th17 cells are preferentially depleted during pathogenic HIV and SIV infection of humans and RMs, with the severity of their depletion being correlated with the levels of systemic immune activation and disease progression [17], [26], [27], [28], [29], [30], [31]. Of note, despite a significant depletion of bulk CD4^+^ T cells from the GI tract, Th17 cells are maintained at a healthy frequency during nonprogressive SIV infections of SMs and AGMs, which are characterized by preservation of mucosal immune function and lack of microbial translocation [8], [26], [28], [32]. Collectively, these published data suggest that lower levels of Th17 cells contribute to the mucosal immune dysfunction and chronic immune activation that distinguish pathogenic from nonpathogenic HIV/SIV infections, and underscore the potential value of novel therapeutic approaches aimed at promoting Th17 cells.

Interleukin (IL)-21 is the most recently identified member of the common γ-chain cytokine family that includes IL-2, IL-4, IL-7, IL-9, and IL-15 [33], [34]. IL-21 is a multifunctional and pleiotropic cytokine mainly produced by activated CD4^+^ T cells, including follicular helper T cells, and NKT cells [33], [34], [35]. IL-21 exerts numerous immune-enhancing and immune-regulatory functions, including (i) favoring the long-term maintenance of functional CD8^+^ T-cells [36], [37], [38], [39], (ii) promoting the differentiation of memory B cells and antibody-secreting plasma cells [40], [41], [42], [43], and (iii) maintaining an adequate pool of mature, fully functional Th17 cells [44], [45], [46]. Reduced plasma levels of IL-21 have been found in HIV-infected individuals [47], [48], and we recently showed that pathogenic SIV-infection of RMs, but not nonpathogenic SIV infection of sooty mangabeys is associated with a significant loss of IL-21-producing CD4^+^ T cells [49]. Since pathogenic HIV/SIV infections are associated with both depletion of Th17 cells and overall reduced availability of IL-21, we hypothesized that *in vivo* administration of IL-21 may result in improved immune function in HIV-infected humans and SIV-infected RM.

To test this hypothesis, in this study we infected twelve RMs with SIV_mac239_ and at day 14 post-infection (p.i.) treated six of them with rhesus rIL-21-IgFc (IL-21 throughout the manuscript). Our results indicate that i*n vivo* administration of IL-21 during acute SIV infection beneficially impacts mucosal immune function by elevating levels of intestinal Th17 cells compared to controls during the treatment period without undesirable effects on viral load. Remarkably, higher levels of intestinal Th17 cells were associated with reduced levels of intestinal immune activation, microbial translocation and systemic inflammation in the chronic phase of infection.

## Results

### Experimental design

**Figure 1:**
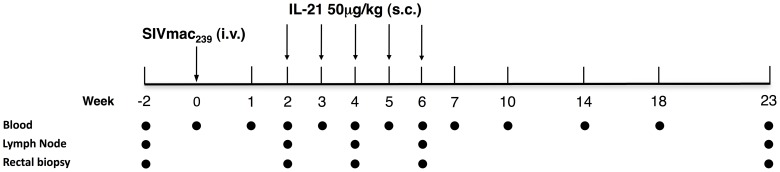
Experimental design of the study. Twelve RMs were infected intravenously (i.v.) with 300 TCID50 SIVmac_239_ (day 0). Starting from day 14 post-infection (p.i.), six animals were treated with 50 µg/kg IL-21 (s.c.) at days 14, 21, 28, 35 and 42 p.i., as indicated by the arrows, with the other six RMs remaining untreated and used as controls. Blood, lymph node and rectal biopsies were collected at numerous experimental points as indicated by the circles. At day 14 p.i. collections of the samples were performed before administration of IL-21, thus this experimental point represents the pre-treatment baseline.

Twelve RMs were infected intravenously (i.v.) with 300 TCID50 SIVmac_239_ (day 0). Starting from day 14 post-infection (p.i.), six animals were treated with five weekly doses of 50 µg/kg IL-21 (s.c.), which represents a dosage similar or lower to those tested in phase I and II clinical trials in humans (IL-21-treated group; orange in graphs) [49]. Specifically, the animals received IL-21 at days 14, 21, 28, 35 and 42 p.i. (Figure 1). The other six SIV-infected RMs remained untreated and were used as controls (control group; black in graphs). The rationale to start therapy in the early phase of infection is that the loss of intestinal Th17 cells in SIV-infected RMs is present within the first few weeks of the initial infection [27]. At numerous experimental points pre- and post-infection, as well as pre- and post-IL-21 treatment, peripheral blood (PB), rectal biopsies (RB), and lymph nodes (LN) were collected from all 12 RMs. All sample collections were performed prior to IL-21 administration at day 14 p.i.; therefore, this experimental point represents the pre-treatment baseline. To study the effect of IL-21 administration on the immune system, samples were collected 3 days after the IL-21 doses. RRq7, in the IL-21-treated group, died at day 75 p.i. Thus, in all graphs the arithmetic mean for IL-21-treated animals includes six RMs up to day 75 and five at later time points.

### IL-21 does not impact on plasma viremia in SIV-infected RMs

**Figure 2:**
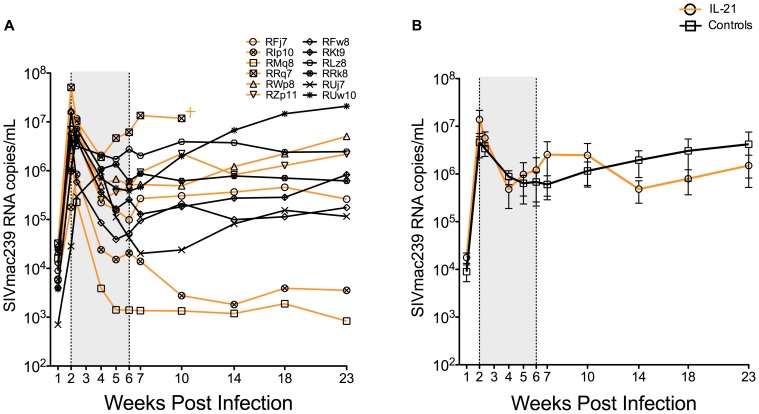
Effects of IL-21 on plasma viremia in SIV-infected RMs. The levels of SIVmac_239_ RNA, expressed as copies/mL, are shown for each individual animal (**A**) and as average (mean±SEM) of IL-21-treated vs. controls RMs (**B**). IL-21-treated animals are depicted in orange, controls in black. Shaded area represents time of IL-21 treatment.

We first examined the effects of IL-21 on the kinetics of SIV plasma viremia. As shown in Figure 2A (individual animals) and 2B (mean±SEM for the two experimental groups), following experimental infection with SIVmac_239_ both groups of RMs experienced a rapid, exponential increase in virus replication that peaked before initiation of IL-21 treatment (at approximately day 14 p.i.) in four out of six IL-21-treated and three out of six control monkeys. Peak viremia occurred on d21 p.i. in two IL-21-treated animals (RMq8 and RFj7) and in three controls (RLz8, RUj7 and RRk8). In the IL-21 treatment group, two animals (RIp10 and RMq8) showed lower post-peak viremia, while a third animal (RRq7), showed viral load remarkably higher than the other RMs already at day 14 p.i., i.e., before IL-21 treatment. This animal did not seroconvert (data not shown) and died at day 75 p.i., thus resembling the phenotype of a rapid progressor [50]. Although the viral load set point was very heterogeneous within each group, there was no significant difference between the two groups at any single experiment time point (Figure 2A,B). Of note, this study was not designed to assess how IL-21 affects survival in SIV-infected RMs, since all animals were started on antiretroviral therapy at wk26 p.i. These limitations notwithstanding, we did not find any significant difference in survival rates, with three out of six animals in both the IL-21 treated group (including the rapid progressor) and the controls group succumbing to AIDS before or immediately after initiation of ART (data not shown).

### Effects of IL-21 on T cell levels in SIV-infected RMs

**Figure 3:**
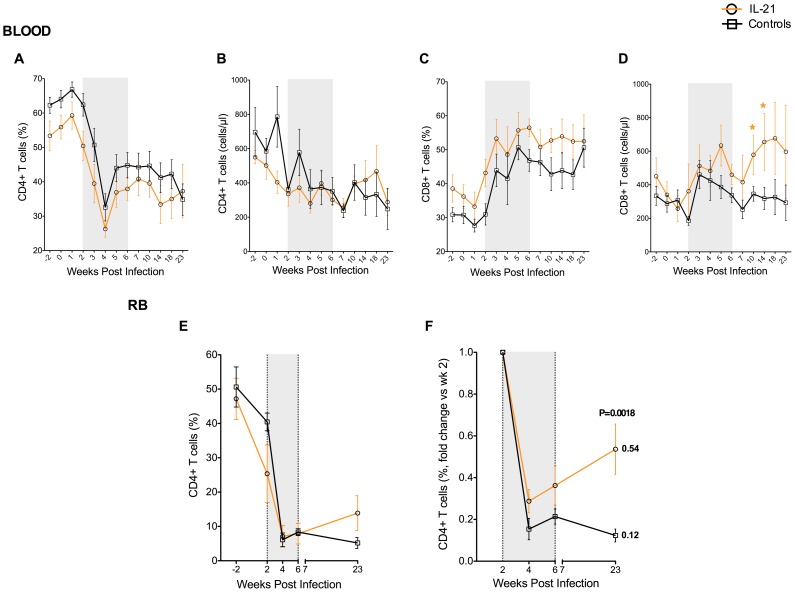
Effects of IL-21 on blood and intestinal T cell levels in SIV-infected RMs. Longitudinal assessment of the percentages (**A, C**) and absolute numbers expressed as cells per µl (**B, D**) of circulating CD4^+^ (**A, B**) and CD8^+^ (**B, D**) T cells in IL-21-treated and controls RMs. (**E–F**) Longitudinal assessment of intestinal CD4^+^ T cells expressed both as fraction of total CD3^+^ T cells (**E**) and as fold change relative to the percentages of CD4^+^ T cells at wk2 (**F**). IL-21-treated animals are depicted in orange, controls in black. Shaded area represents time of IL-21 treatment. Averaged data are presented as mean ± SEM.

We next assessed the effects of IL-21 on the levels of T cells by determining the relative frequencies (in PB, LN, and RB) and absolute numbers (in PB) of CD4^+^ and CD8^+^ T cells in the sampled anatomical sites. The kinetics of the percentages (Figure 3A) and absolute number (expressed as cells per µl, Figure 3B) of circulating CD4^+^ T cells were overall very similar in IL-21-treated and control RMs, with no significant differences among the two groups at any single experimental points. Similar to CD4^+^ T cells, the percentages of circulating CD8^+^ T cells were also not affected by IL-21 treatment (Figure 3C), although their numbers were significantly higher in IL-21-treated RMs than controls at wks 10 (P = 0.04) and 14 (P = 0.03) p.i., when a mixed linear-effects model was used to assess the longitudinal significance of differences between groups (Figure 3D). Similarly, IL-21 treatment did not have a significant impact on T cells in the LN, with the overall percentages of CD4^+^ and CD8^+^ T cells remaining very similar between IL-21-treated and controls animals at all tested experimental points (data not shown).

We next determined the effects of IL-21 treatment on intestinal T cells by measuring the levels of CD4^+^ and CD8^+^ T cells in RBs. This part of the study is important since mucosal CD4^+^ T cells are severely depleted during the acute phase of HIV and SIV infections, and the severity of this depletion correlates with disease progression [14], [15], [16], [51]. Consistent with previous findings, SIV infection in RMs was associated with a rapid and severe depletion of CD4^+^ T cells in the RB (Figure 3E). Interestingly, the percentages of intestinal CD4^+^ T cells increased between wk6 and wk23 p.i. (7.90±3.00 vs. 13.89±6.51) in IL-21-treated RMs, but decreased (8.35±0.97 vs. 5.20±1.58) during the same time period in control animals (Figure 3E). However, percentages of CD4^+^ T cells were not statistically significant at any single experimental time point, and the two animals that better control viral load were those with higher percentages of CD4^+^ T cells post-infection (Figure S1A).

The difference in the levels of intestinal CD4^+^ T cells between the two groups is more evident when the results are represented as fold change relative to the percentages of CD4^+^ T cells at wk2, thus accounting for the variability of the intestinal CD4^+^ T cell depletion during the first two weeks of infection (Figure 3F and Figure S1B). This analysis showed that at wk23 p.i. IL-21-treated RMs maintained a significant fraction of the total CD4^+^ T cells that were present at wk2 p.i. (fold change: 0.54±0.12; Figure 3F), while in control animals the levels of CD4^+^ T cells at wk23 dropped to 0.12±0.03 of those present at wk2 p.i. (fold change), with this difference being statistically significant (Figure 3F; P = 0.0018). Of note, this effect of IL-21 was specific for CD4^+^ T cells, since no significant variations were found in the percentages of intestinal CD8^+^ T cells (data not shown).

Together with those of Figure 2, these data indicate that IL-21 treatment in the acute phase of SIV infection of RMs has a limited impact on plasma viremia and on the levels of circulating T cells, but appears to be associated with a better preservation of intestinal CD4^+^ T cells.

### IL-21 treatment induces perforin and granzyme B in total and virus specific CD8^+^ T Cells of SIV-infected RMs

**Figure 4:**
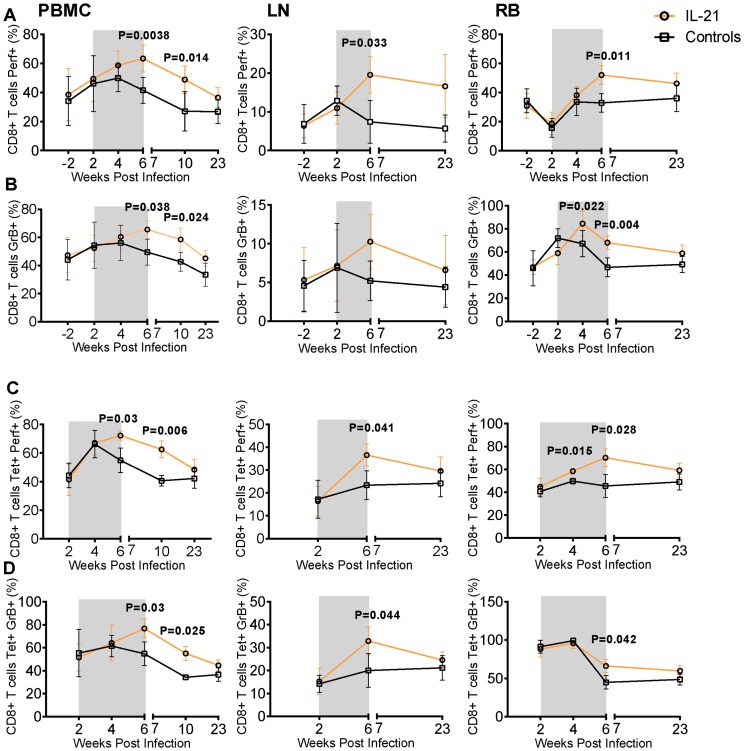
IL-21 administration during acute SIV infection in RMs induces perforin and GrB in T cells of various anatomical sites. Intracellular expression of perforin and GrB was analyzed in total CD8^+^ T cells and Tetramer^+^ CD8^+^ T cells from PBMC, LN and RB by flow cytometry. Longitudinal assessment of the mean frequencies of (**A**) perforin and (**B**) GrB in gated live CD3^+^CD8^+^ T cells. Longitudinal assessment of the mean frequencies of (**C**) perforin and (**D**) GrB in A*01CM9-Gag_181–189_ Tetramer^+^ CD8^+^ T cells. IL-21-treated animals are depicted in orange, controls in black. Shaded area represents time of IL-21 treatment. Averaged data are presented as mean ± SEM.

Previous studies showed that IL-21 is needed for long-term maintenance of functional CD8^+^ T cells in LCMV-infected mice [37], [52], [53], [54] and stimulates production of cytotoxic molecules in human CD8^+^ T cells *in vitro* [55]. Based on these data, we sought to determine by flow cytometry the intracellular levels of perforin and granzyme B (GrB) in total and SIV-specific (gag-tetramer^+^) CD8^+^ T cells, as well as their maturation subsets. Perforin and GrB analysis was performed at wks-2 (pre-infection), 2 (pre-IL-21 administration), 4 and 6 (during IL-21 treatment), 10 and 23 p.i. in PBMC; at wks-2, 2, 4, 6 and 23 in RB and at wks-2, 2, 6 and 23 in LN. As shown in Figure 4, no significant differences were noted for baseline levels of perforin or GrB between IL-21-treated and control RMs in all studied anatomical sites. In comparison to control animals, IL-21-treated RMs manifested significant increases in the frequency of perforin positive CD8^+^ T cells in PBMC (P = 0.0038), LN (P = 0.033) and RB (P = 0.001) at wk6 p.i., i.e., after the final IL-21 dose (Figure 4A). Interestingly, the percentage of CD8^+^perforin^+^ T cells in PBMC was still significantly higher in IL-21-treated than control RMs at wk10 p.i., i.e. approximately one month after the last dose of IL-21 (Figure 4A; P = 0.014). Analysis of GrB expression showed a pattern very similar to that found for perforin, with the percentage of CD8^+^GrB^+^ T cells being significantly higher in IL-21-treated RMs at wks6 and 10 in PBMC (P = 0.038 and 0.024, respectively) and at wks4 and 6 in RB (P = 0.022 and 0.0044), despite very similar levels before starting treatment (Figure 4B). Differently from perforin, no differences were found in the percentages of CD8^+^GrB^+^ T cells in LN. The increase in perforin and GrB expression was specific for both central (CD28^+^CD95^+^CCR7^+^) and effector memory (CD28^−^CD95^+^CCR7^−^) CD8^+^ T cell subsets of PBMC, LN and RB (data not shown). We then investigated the ability of IL-21 to induce perforin and GrB in virus specific (SIV gag tetramer^+^) CD8^+^ T cells in the six *Mamu*-A*01 animals (three in each group) included in the study. IL-21 did not change the frequencies of CD8^+^tet^+^ cells, which increased similarly in both groups following infection (data not shown). However, similar to the observations in total CD8^+^ T cells, IL-21 significantly increased the percentages of SIV-specific CD8^+^perforin^+^ T cells at wk4 p.i. in RB (P = 0.015), at wk6 in PBMC (P = 0.03), LN (P = 0.041), and RB (P = 0.028), and at wk10 in PBMC (P = 0.006) compared to control animals (Figure 4C). Importantly, only in IL-21-treated RMs were the frequencies of SIV-specific CD8^+^perforin^+^ T cells in PBMC (P = 0.0134), LN (P = 0.036) and RB (P = 0.042) significantly higher at wk6 than wk2 p.i., thus confirming the ability of IL-21 to specifically induce perforin production in virus specific CD8^+^T cells from different anatomical sites (Figure 4C). Similar results were found for GrB, with the frequencies of SIV-specific CD8^+^GrB^+^ T cells being significantly higher in IL-21-treated animals than controls at wk6 in PBMC, LN and RB, and at wk10 in PBMC (Figure 4D).

Finally, we investigated the effects of IL-21 administration on perforin and GrB production by CD4^+^ T cells. The effects on perforin induction were limited, with the percentages of CD4^+^perforin^+^ T cells in PBMC, LN and RB remaining very similar in IL-21-treated and controls animals throughout the study (Figure S2A). In contrast to perforin expression, the frequencies of CD4^+^GrB^+^ T cells were significantly increased in IL-21-treated RMs at wk6 in PBMC, LN and RB, and sustained through wk10 in PBMC (Figure S2B).

These data underscore the ability of IL-21 to enhance and/or maintain the expression of the T cell cytotoxic granules perforin and GrB in total and virus specific CD8^+^ T cells in various anatomical sites of SIV-infected RMs.

### IL-21 treatment increases the frequencies of memory B cells in SIV-infected RMs

Among its immunologic functions, IL-21 has been shown to promote differentiation of antigen-stimulated B cells into memory B cells and antibody-secreting plasma cells [40], [41], [42], [43]. For this reason, we then sought to investigate if administration of IL-21 to SIV-infected RMs could impact the levels of the different B cell maturation subsets, which quantification was performed using a combination of antibodies for CD3, CD20, CD21, CD27, and IgD [56], [57], [58]. Consistent with previous reports [56], [58], animals in both groups showed a significant decrease in the blood frequencies of memory B cells (CD3^−^CD20^+^CD21^hi^CD27^+^) at wk2 p.i. (Figure S3A). While in the percentages of memory B cells further decline at wk6 and wk10 p.i. in control animals, their levels stabilize or even increase in IL-21-treated RMs. Indeed, the percentages of circulating memory B cells were significantly higher in IL-21-treated than control animals at wk6 and wk10 p.i. (P = 0.004 for both time points). At the same experimental points, IL-21-treated RMs also showed higher frequencies of switch memory B cell (CD3^−^CD20^+^CD21^hi^CD27^+^IgD^−^) when compared to controls (Figure S3B; wk6 P = 0.016; wk10 P = 0.042). Finally, we investigated the effects of IL-21 treatment on the levels of anti-SIV antibodies. Total levels and kinetics of anti-SIV antibodies were virtually indiscernible between IL-21 treated and control RMs (Figure S3C).

All together, these findings suggest IL-21 treatment impacts the homeostasis of the different B cell maturation subsets by increasing the frequency of memory B cells while reducing their levels of activation.

### IL-21 treatment increased the levels of intestinal Th17 cells in SIV-infected RM

**Figure 5:**
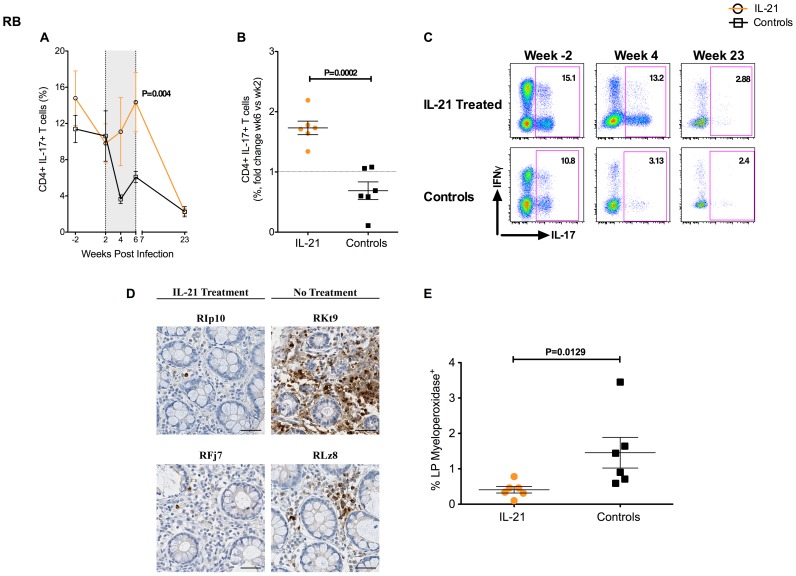
Effects of IL-21 on intestinal Th17 cells in SIV-infected RMs. Intestinal Th17 cells were identified by flow cytometry as CD4^+^ T cells that produce IL-17 after brief *in vitro* stimulation with PMA and ionomycin. (**A**) Longitudinal assessment of intestinal CD4^+^IL-17^+^ T cells in IL-21-treated and controls RMs. Shaded area represents time of IL-21 treatment. (**B**) Fold change in the percentages of Th17 cells between wk6 (end of treatment) and wk2 (pre-treatment) in IL-21-treated and controls RMs. (**C**) Representative co-staining of IL-17 and IFN-γ within intestinal CD4^+^ T cells in one IL-21-treated (top plots) and one control RM (bottom plots) at weeks -2 (pre-infection), 4 (during the IL-21 treatment) and 23 p.i. (**D**) Rectal mucosa tissues were stained for myeloperoxidase (brown) as a marker for PMNs. Representative images (200×) in two IL-21-treated animals (left panels) and two controls (right panels) at wk6 p.i. (**E**) Random high power 400× images of gut lamina propria (LP) were taken and the percent area staining for myeloperoxidase was determined at wk6 p.i. in IL-21-treated and control RMs. IL-21-treated animals are depicted in orange, controls in black. Averaged data are presented as mean ± SEM.

IL-21 is a key Th17-inducing cytokine, as it is able to promote both development and survival of these cells [44], [45], [46], [59]. Based on these findings, we next assessed the effects of IL-21 treatment on the levels of intestinal Th17 cells, identified by flow cytometry as CD4^+^IL-17^+^ T cells after brief *in vitro* stimulation with PMA and ionomycin. IL-21-treated SIV-infected RMs showed levels of Th17 cells consistently higher compared to those found in controls throughout the entire treatment period (Figure 5A). Remarkably, after an initial depletion from day 0 to day 14 p.i., the percentage of Th17 cells stabilized or even increased in IL-21-treated animals up to wk6 p.i., in marked contrast to the rapid and severe depletion of Th17 cells observed in controls (Figure 5A; P = 0.004 at wk6). The efficacy of IL-21 in preserving intestinal Th17 cells is particularly clear when the levels of these cells are expressed as fold change compared to pre-treatment (i.e., wk2 p.i.). As shown in Figure 5B, the fold change in the percentages of Th17 cells between wk6 (end of treatment) and wk2 was significantly higher for IL-21-treated than controls RMs (1.73±0.11 vs. 0.69±0.15; P = 0.0002). Furthermore, better preservation of Th17 cells was noted in all six SIV-infected RMs, independent of their viral load or severity of intestinal CD4^+^ T cell depletion (Figure 5B). Despite this beneficial effect of IL-21 treatment observed at wk6, the levels of intestinal Th17 cells in IL-21-treated RMs declined to levels comparable to those found in control animals by wk23 p.i., i.e., 4 months after the last administration of IL-21 (Figure 5A). These data suggest that the higher levels of intestinal Th17 cells were specifically related to IL-21 treatment and that its beneficial effects on Th17 cells were transient. Of note, the levels of Th17 cells were overall similar in blood (Figure S4) and LN (data not shown) of treated and control RMs. Moreover, we found that IL-21 acted selectively on Th17 cells, since blood and intestinal CD4^+^IFN-γ^+^ or CD4^+^IL-2^+^ T cells were depleted in a very similar way in IL-21-treated and untreated RMs (Figure S4). A representative co-staining of IL-17 and IFN-γ within intestinal CD4^+^ T cells is shown in one IL-21-treated and one control RM at wks-2 (pre-infection), 4 (during the IL-21 treatment) and 23 p.i. (Figure 5C). Consistent with an effect of IL-21 on Th17 cells, plasma levels of IL-22 were higher in IL-21-treated than control animals at wk6 post infection (fold change wk6 vs. wk2: 5.6±1.82 vs. 1.02±0.39; P = 0.056; Figure S5).

Finally, in order to assess the extent of the loss of integrity of the epithelial barrier of the GI tract, we performed immunohistochemistry analysis for the polymorphonuclear neutrophil (PMN) infiltration in rectal biopsy tissues of IL-21-treated and control animals at wk6 post-infection (Figure 5D). PMN migration along the intestinal epithelia is the hallmark of intestinal inflammation and has been correlated with the degree of intestinal epithelial barrier dysfunction in SIV-infected RMs [60]. PMN activation leads to accumulation of myeloperoxidase (MPO)+ PMNs adjacent to epithelial lesions, reflecting a tissue response to loss of epithelial integrity. Interestingly, levels of MPO+ PMNs in the lamina propria were significantly lower in IL-21-treated than control animals (P = 0.0129; Figure 5E), thus indicating better maintenance of gut integrity following IL-21 treatment.

Together, these findings indicate that administration of IL-21 during early SIV-infection of RMs significantly improved the preservation of intestinal Th17 cells and the integrity of the mucosal barrier.

### IL-21 treatment reduces intestinal T cell proliferation, microbial translocation and systemic activation/inflammation in SIV-infected RMs

**Figure 6:**
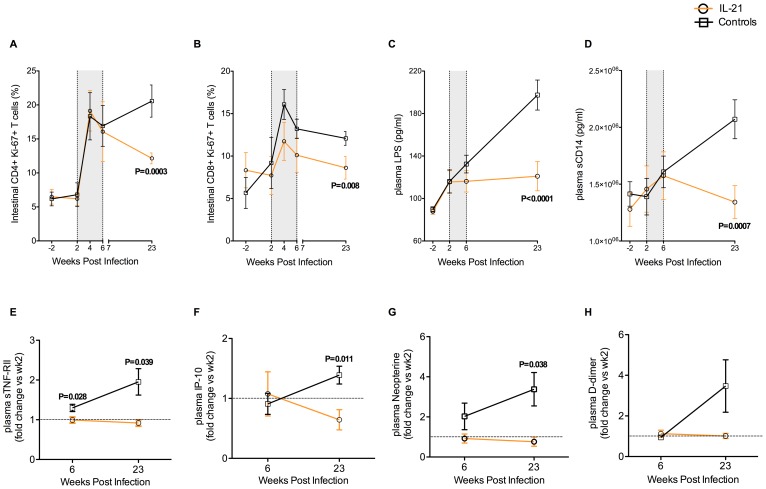
Effects of IL-21 on intestinal T cell proliferation, microbial translocation and systemic inflammation in SIV-infected RMs. (**A, D**) Longitudinal assessment of the fraction of intestinal (**A**) CD4^+^Ki-67^+^ and (**B**) CD8^+^Ki-67^+^ T cells and of plasma levels (pg/ml) of (**C**) LPS and (**D**) sCD14 in IL-21-treated and controls RMs. Shaded area represents time of IL-21 treatment. (**E–H**) Longitudinal assessment of plasma levels of (**E**) sTNF-RII (pg/ml), (**F**) IP-10 (pg/ml), (**G**) Neopterine (nmol/L) and (**H**) D-dimer (ng/ml) in IL-21-treated and controls RMs. Data in (**E–H**) are shown as fold change variation at wk6 (end of treatment) or wk23 (end of study) as compared to wk2 (pre-treatment) p.i. IL-21-treated animals are depicted in orange, controls in black. Averaged data are presented as mean ± SEM.

Several studies have suggested that a preferential loss of intestinal Th17 cells is a key mechanism responsible for the mucosal immune dysfunction and associated chronic immune activation that are typical of pathogenic HIV/SIV infections [17], [26], [27], [28]. Indeed, in both HIV-infected humans and SIV-infected RMs, the levels of Th17 cells and those of proliferating CD4^+^ T cells are strictly associated [17], [28], [29]. To investigate if the increased levels of intestinal Th17 cells induced by IL-21 are associated with limited intestinal T cell activation and/or proliferation we compared the percentages of CD4^+^ or CD8^+^ T cells expressing the activation markers HLA-DR and CD69 or the proliferation marker Ki-67 in the RBs of IL-21-treated and control RMs. While no significant differences were found at any experimental time point in the levels of CD4^+^ or CD8^+^ T cells expressing HLA-DR or CD69 (data not shown), the percentages of CD4^+^Ki-67^+^ T cells were significantly lower in IL-21-treated as compared to control RMs at wk23 p.i. (Figure 6A; 12.14±0.80 vs. 20.57±2.37; P = 0.0003). Remarkably, limited proliferation of intestinal CD4^+^ T cells was consistently found at wk23 in all IL-21-treated RMs (Figure S6A for individual values). IL-21 treatment was also effective in limiting proliferation of intestinal CD8^+^ T cells, with the percentage of CD8^+^Ki-67^+^ T cells at wk23 p.i. being significantly lower in IL-21-treated than in controls (Figure 6B; 8.61±1.33 vs. 12.1±0.81; P = 0.0080; Figure S6B for individual values). To further investigate the effect of IL-21 on the integrity of the intestinal mucosa, we measured the plasma levels of LPS and sCD14, which are commonly used as surrogate markers of microbial translocation from the intestinal lumen to systemic circulation. As previously described for SIV-infected RMs, in the six control animals microbial translocation increased progressively during the transition from acute to chronic infection, with the plasma levels of LPS (P = 0.0012) and sCD14 (P<0.0001) at wk23 being significantly higher than those at wk2 p.i. (Figure 6C–D). In contrast, in IL-21-treated RMs, plasma levels of LPS and sCD14 throughout the study remained very similar to those present before initiation of IL-21 treatment, with plasma levels of LPS and sCD14 at wk23 significantly lower than those of control animals (Figure 6C–D; LPS: 121±13.7 vs. 197.4±14.2; P<0.0001; sCD14: 1,342,000±145,029 vs. 2,071,000±171,117; P = 0.0007; Figure S6C–D for individual values). To analyze the magnitude of the effects of IL-21 treatment on sCD14 and LPS levels, we then compared the levels of these two markers at baseline (before infection) versus at wk23 post-infection. At wk23, in IL-21-treated animals, sCD14 levels were similar to those seen at baseline, while LPS levels were still higher than baseline, although significantly lower than in controls and remarkably stable (in contrast to a progressive increase in controls) from wk4 up to the end of the study (Figure 6C–D).

Finally, we sought to investigate if these reduced levels of microbial translocation observed in IL-21-treated SIV-infected RMs were associated with lower levels of systemic T cell activation and/or proliferation. Using the same markers described for the GI tract, we did not find any significant difference between IL-21-treated and control RMs in the percentages of circulating CD4^+^ or CD8^+^ T cells expressing HLA-DR, CD69, Ki-67 or PD-1 at any of the studied experimental points (data not shown and Figure S7). In addition to cellular markers of immune activation, we measured the fold change variation compared to pre-treatment (wk2 p.i.) in several soluble markers of inflammation. As shown in Figure 6E–H, the fold change in plasma levels of sTNF-RII (wk6: P = 0.028; wk23: P = 0.039), IP-10 (wk23: P = 0.011), and neopterine (wk23: P = 0.038) was significantly lower for IL-21-treated than controls RMs. D-dimer levels were also lower in IL-21 treated than control animals, although this difference was not statistically significant (Figure 6H).

Together with those of Figure 5, these data indicate that in IL-21-treated RMs a better preservation of intestinal Th17 cells in early infection is associated with reduced levels of intestinal T cell proliferation, microbial translocation and systemic inflammation in the chronic stage of infection.

### Preservation of intestinal Th17 cells is associated with limited intestinal T cell proliferation and microbial translocation in IL-21-treated SIV-infected RMs

**Figure 7:**
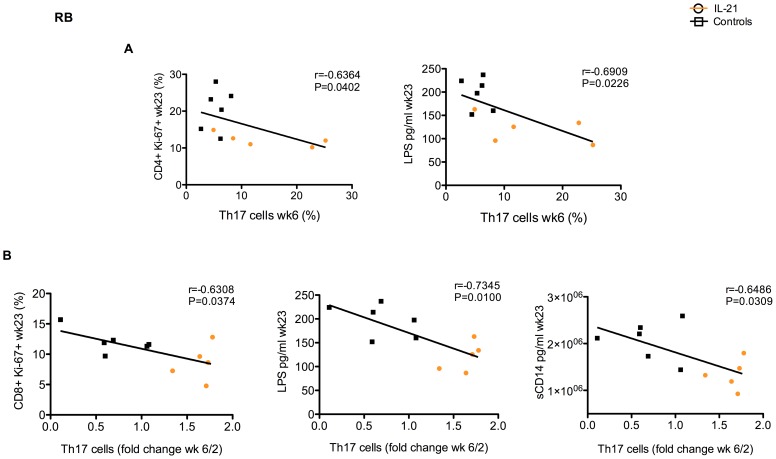
Levels of intestinal Th17 cells are associated with limited intestinal T cell proliferation and microbial translocation in SIV-infected RMs. (**A**) The percentage of intestinal CD4^+^IL-17^+^ T cells at wk6 negatively correlated with the percentages of intestinal CD4^+^Ki-67^+^ T cells (left panel) and the plasma levels of LPS (right panel) at wk23 p.i. (**B**) Fold change in the percentages of intestinal CD4^+^IL-17^+^ T cells between wk6 and wk2 negatively correlated with the percentages of intestinal CD8^+^Ki-67^+^ T cells (left panel), and the plasma levels of LPS (middle panel) and sCD14 (right panel). Pearson product-moment correlation coefficients were utilized to estimate linear associations for normally distributed data (Figure 7B) and Spearman rank correlation coefficients (Figure 7A) were used for skewed and other non-normal distributions. IL-21-treated animals are depicted in orange, controls in black.

To further investigate the association between Th17 cells, mucosal immune activation, and microbial translocation, we next investigated the relationship between the levels of intestinal Th17 cells, those of proliferating T cells, and the markers of microbial translocation, LPS and sCD14. Consistent with the known role of Th17 cells in promoting mucosal immunological and physical integrity, we found that the percentages of intestinal Th17 cells at wk6 negatively correlated with the percentages of intestinal CD4^+^Ki-67^+^ T cells (r = −0.6364; P = 0.0402) and the plasma levels of LPS (r = −0.6909; P = 0.0226) at wk23 p.i. (Figure 7A). Furthermore, when expressed as fold change compared to wk2, the levels of intestinal Th17 cells at week 23 p.i. were inversely correlated to the percentage of intestinal CD8^+^Ki-67^+^ T cells (r = −0.6308; P = 0.0374) and the plasma levels of LPS (r = −0.7345; P = 0.0100) and sCD14 (r = −0.6486; P = 0.0309) (Figure 7B). Of note, no significant correlations were found between the levels of intestinal Th17 cells at wk6 and those of viral load at wk6 or at wk23 (data not shown).

Collectively, these data indicate a strong association between IL-21-dependent preservation of intestinal Th17 cells and limited levels of intestinal T cell proliferation and microbial translocation in IL-21-treated SIV-infected RMs.

## Discussion

In this study, we tested the hypothesis that exogenous *in vivo* administration of IL-21, a cytokine that possesses numerous unique immunological properties, is beneficial in the context of pathogenic lentiviral infections. To test this hypothesis, we used the nonhuman primate model of SIV infection in rhesus macaques (RMs) and treated the animals with IL-21 during the acute phase of infection (i.e., starting at day 14). The rationale for this study was based on several findings. First, a large body of evidence has shown that IL-21 is a key factor in regulating central processes that are defective and/or compromised in HIV/SIV infected humans and RMs. These processes include differentiation and homeostatic expansion of Th17 cells [44], [45], [46], [59], long-term maintenance of functional CD8^+^ T cells [36], [37], [38], [39], and differentiation of memory B cells and Ab-secreting plasma cells [40], [41], [42], [43]. Second, the overall availability of IL-21 is reduced in both HIV-infected humans [47], [48] and SIV-infected RMs [49], while high level of HIV-specific CD4^+^IL-21^+^ and CD8^+^IL-21^+^ T cells are present in the rare subset of HIV-infected individuals who are able to spontaneously control HIV replication without treatment [38], [61]. Third, we recently described in a cross-sectional study of SIV-infected RMs a strong association between the levels of IL-21-producing CD4^+^ T cells and those of Th17 cells, with a significant portion of the variability in Th17 cells being dictated by variability in CD4^+^IL-21^+^ T cells [49]. Fourth, we previously showed that IL-21 induces the expression of the cytotoxic molecules perforin and granzyme B in CD8^+^ T cells and NK cells *in vitro* in chronically HIV-infected individuals [55], [62] and *in vivo* in a pilot study where IL-21 was administered to late-stage disease SIV-infected RMs [57]. Lastly, IL-21 is currently used in phase I and II clinical trials in renal cell carcinoma, melanoma and non-Hodgkin's lymphoma with an overall good safety profile and encouraging single agent activity [49], [63], [64], [65]. Specifically, immunological analysis of these phase I studies showed that IL-21 treatment increased the mRNA levels for IFN-γ, perforin, granzyme B and granzyme A in CD8^+^ T cells and NK cells and the expression of ICOS and chemokine receptors (CCR5, CXCR6 and CXCR3) in both CD4^+^ and CD8^+^ T cells. According to these studies, the immunologic mechanisms of the antitumor action of IL-21 involve augmented NK cell and CD8^+^ T cell cytotoxicity and increased migratory properties of these cells to sites of inflammation [63], [64], [65].

The key findings of the current study are the following: (i) IL-21 treatment was safe and its administration during the acute phase of infection did not increase plasma viral load, systemic T cell activation or levels of the T cell inhibitory molecule PD-1; (ii) IL-21 significantly increased the expression of Perforin and GrB in total and virus specific CD8^+^ T cells in blood, LN, and rectal mucosa; (iii) IL-21 treatment increased the frequencies of memory B cells; (iv) IL-21 increased the levels of intestinal Th17 cells and the integrity of the mucosal barrier in the early infection; and (v) preservation of intestinal Th17 cells was associated with increased preservation of intestinal CD4^+^ T cells, reduced proliferation of intestinal CD4^+^ and CD8^+^ T cells, limited microbial translocation and systemic inflammation in the chronic phase of infection. Table S1 summarizes the experimental time points in which specific parameters were significantly different between IL-21-treated and control animals.

As mentioned above, IL-21 has been implicated as a key factor regulating the differentiation and maintenance of Th17 cells, a CD4^+^ T cell subset crucial for mucosal immunity [44], [45], [46], [59]. In HIV-infected humans and SIV-infected RMs, depletion of mucosal CD4^+^ T cells preferentially involves Th17 cells [17], [26], [27], [28]. It is believed that Th17 cell depletion favors a breakdown of the physical and/or biological mucosal barrier with translocation of bioactive microbial products from the intestinal lumen to the systemic circulation, thus contributing to the establishment of the high levels of chronic immune activation [2], [19]. The evidence that mucosal Th17 cells are preserved in SIV-infected sooty mangabeys, which maintain mucosal integrity and avoid microbial translocation and chronic immune activation, is consistent with a key role of these cells in controlling mucosal integrity and immune activation. The current study shows that IL-21 treatment results in better preservation of intestinal Th17 cells in SIV-infected RMs. Intriguingly, after the initial depletion of Th17 cells in the first 2 weeks p.i., IL-21-treated RMs did not show any further loss of Th17 cells, with the percentage of these cells remaining stable until wk6 p.i.. These kinetics were remarkably different from the severe and progressive depletion of Th17 cells experienced by control RMs in the same time frame. Of note, the beneficial effects of IL-21 were specific for intestinal Th17 cells, since CD4^+^IFN-γ^+^ and CD4^+^IL-2^+^ T cells were not affected by the treatment. When this study was started, IL-22 antibodies cross-reactive for RMs were not commercially available, and therefore we could not determine if the Th17 cells induced by IL-21 treatment were able to produce IL-22 in addition to IL-17. However, we could measure the plasma levels of IL-22 and found a five-fold increase (p = 0.056) in the plasma levels of IL-22 at wk6 in IL-21-treated animals compared to controls. To the best of our knowledge, this is the first study showing an intervention able to selectively preserve Th17 cells *in vivo* in SIV-infected RMs. Unfortunately, the effects of IL-21 on Th17 cells were temporary, as four months post-treatment the depletion of Th17 cells was comparable in IL-21-treated RMs and control animals. Overall these data underscore the need for a more prolonged treatment with IL-21 *in vivo* and for further studies aimed at optimizing the regimen for IL-21 administrations.

Depletion of intestinal Th17 cells contributes to the mucosal immune dysfunction and associated immune activation in pathogenic HIV/SIV infections with a reported association between the levels of Th17 cells and those of proliferating CD4^+^ T cells [17], [28], [29]. Consistent with a mechanistic link between mucosal Th17 cells and immune activation, in IL-21-treated RMs a better preservation of intestinal Th17 cells was associated with significantly lower levels of (i) proliferating intestinal CD4^+^ and CD8^+^ T cells; (ii) microbial translocation, as assessed by plasma levels of LPS and sCD14; and (iii) inflammation, as assessed by quantification of several plasma markers. In addition, the lower levels of neutrophil infiltration, commonly used as surrogate for the gut barrier integrity [60], indicate a better preserved intestinal epithelial barrier in IL-21-treated animals than controls. These data are of interest considering that impaired mucosal integrity is a key contributor to HIV-associated chronic immune activation [2], [9], [10]. Surprisingly, in IL-21-treated SIV-infected RMs, limited microbial translocation and reduced levels of soluble markers of inflammation did not translate into lower levels of T cell activation or proliferation in blood. These data suggest that, at least in this study, plasma levels of LPS and sCD14 are more associated with systemic inflammation than with peripheral T cell activation/proliferation. It is also possible, however, that follow up was not long enough to observe effects of IL-21 on systemic immune activation, since animals started antiretroviral therapy at seven months p.i.

IL-21 may regulate two other immunological functions that are compromised in pathogenic HIV/SIV infections, i.e., the cytolytic potential of CD8^+^ T cells [36], [37], [38], [39] and the differentiation of Ag-stimulated B cells into memory B cells and antibody (Ab)-secreting plasma cells [40], [41], [42], [43]. Our results indicate that, in comparison to control animals, IL-21-treated SIV-infected RMs experience a significant increase in the frequency of perforin and granzyme positive CD8^+^ T cells for up to one month after IL-21 treatment. Of note, the increase in perforin and GrB expression was confirmed also for SIV-specific CD8^+^ T cells, and in total and virus-specific CD8^+^ T cells of various anatomical locations. In our examination of B cells, we found that IL-21 had an impact on the levels of the different B cell maturation subsets, with treated animals showing higher frequencies of CD20^+^CD21^hi^CD27^+^ memory B cells and CD20^+^CD21^hi^CD27^+^IgD^−^ switch memory B cells as compared to control animals up to one month after the last IL-21 administration. Our current data on the *in vivo* effects of IL-21 administration on the cytotoxic potential of T cells and expansion of memory B cells is consistent with the findings that we previously reported during chronic SIV infection in RMs [57]. The originality of the data generated in the current study on the cytotoxic potential of T cells and expansion of memory B cells includes (i) the usage of a different form of rIL-21 as well as a different dose and route of administration, (ii) IL-21 treatment being performed during acute SIVmac239 infection, (iii) expression of cytotoxic molecules in SIV-specific CD8^+^ T cells, (iv) cytotoxic potential of T cells and expansion of memory B cells determined in various anatomical sites, including rectal mucosa.

While IL-21 treatment had a beneficial impact on CD8^+^ T cell functions, viral loads were not significantly different between IL-21 treated and control RMs. Reduction of viral load may be very difficult to achieve with any single cytokines in particular during the acute phase of infection. It is also possible that IL-21 had diverse, multiple effects on viral load that counterbalance each other, thus resulting in a lack of variation in viral load. This finding led us to conclude that better preservation of intestinal Th17 cells, rather than changes in CD8^+^ T cells and B cells, is the main mechanism by which IL-21 impacts mucosal immune functions. Consistent with this interpretation, and despite the limited number of samples, higher levels of intestinal Th17 cells at wk6 were associated with lower levels of intestinal T cell proliferation and microbial translocation at wk23 p.i.

A precise and extensive *in situ* quantitation of Th17 cells as numbers/unit of tissue by immunohistochemistry was not possible in the relatively small amount of tissues that can be collected during a longitudinal study in RMs. Furthermore, a mechanistic investigation on how IL-21 increases levels of Th17 cells is beyond the scope of the current body of work. Indeed, additional studies are needed to determine which stage of the Th17 cell differentiation pathway is mainly affected by IL-21, and why the treatment was more effective for intestinal Th17 cells than blood or LN-resident Th17 cells. In particular, these studies should investigate several non-mutually exclusive mechanisms, including (i) IL-21-mediated increase in the homing of blood and LN-resident Th17 cells to mucosal sites; (ii) IL-21-mediated increase in the levels of Th17 cell precursors; (iii) IL-21-mediated differentiation of Th17 cell precursors in fully mature Th17 cells; (iv) IL-21-mediated increase in the proliferation and/or survival of fully mature Th17 cells; and (v) IL-21-mediated decrease in HIV/SIV susceptibility by Th17 cells. Finally, it will be important to expand our investigation in an experimental setting that is more directly relevant to HIV infection in humans, i.e. IL-21 treatment performed in the chronic phase of SIV infection and in association with ART.

In conclusion, IL-21 treatment in SIV-infected RMs increases the levels of intestinal Th17 cells, reduces virus-associated mucosal immune dysfunction and chronic immune activation, and limits microbial translocation and systemic inflammation. These findings provide rationale for further preclinical studies aimed at exploring IL-21 as a potential immune-based intervention to be used in the context of ART during the chronic phase of infection.

## Materials and Methods

### Ethics statement

All animal experimentations were conducted following guidelines established by the Animal Welfare Act and the NIH for housing and care of laboratory animals and performed in accordance with Institutional regulations after review and approval by the Institutional Animal Care and Usage Committees (IACUC; Permit Number: 2001973) at the Yerkes National Primate Research Center (YNPRC). All efforts were made to minimize suffering.

### Animals and SIV-infection

Twelve female RMs, all housed at the Yerkes National Primate Research Center, Atlanta, GA, were included in the study. All animals were *Mamu*-B*08 and B*17 negative, while six of them were *Mamu*-A*01 positive (RIp10, RFj7, RZp11, RUj7, RUw10, RKt9). The 12 RM were randomized in two groups (Group 1: IL-21-treated; Group 2: controls) of six animals based on age (Group. 1: 8±2.45; Group. 2: 8±1.42), weight (7.85±2.19 vs. 7.58±1.08) and *Mamu*-A*01 status (three in each group). All 12 animals were infected intravenously (i.v.) with 300 TCID50 SIVmac239 (day 0). Starting from day 14 p.i., the six animals of group 1 were treated with five weekly doses (subcutaneous) of 50 µg/kg rhesus rIL-21-IgFc (IL-21). The six RMs of group 2 remained untreated and used as controls (Figure 1). Peripheral blood (PB), rectal mucosa (RB) and lymph node (LN) biopsies were collected at numerous experimental points prior- (day -14) and post-infection, before (day 14 p.i.), during (day 14–42) and post IL-21 treatment (Figure 1).

### Production and testing of rhesus rIL-21-IgFc

Rhesus rIL-21-IgFc fusion protein was produced in the Drosophila S2 system by the Resource for Nonhuman Primate Immune Reagents with rmamuIL-21 fused to a macaque IgG2 Fc mutated to prevent binding to complement or Fc receptors similar to a PD-1-IgFc reported before [66]. IL-21-Fc was purified by affinity chromatography to Protein-G sepharose to >95%, dyalized against PBS and tested for sterility and the potential presence of residual endotoxin. Content and bioactivity of the cytokine batches were verified by EIA (B–D antibody pairs J148-1134 and I76–539) and its capacity to upregulate the expression of perforin and granzyme B in PBMC and LN cells of healthy and SIV-infected Rhesus macaques *in vitro* and by administration to healthy RMs [57]. The rationale for the dose utilized was based on a series of studies by our laboratories on the administration and biological effect of gamma chain stimulating cytokines IL-2, IL-7 and IL-15 [67], [68], [69] and on our previous dose escalation study using E coli produced recombinant mamuIL-21 in which maximal upregulation of perforin was obtained above 10 µg/kg [57]. The rationale for using rIL-21-IgFc fusion protein and not rIL-21 as in our previous study [57] was based on our data indicating that (i) the biological activity based on protein content was comparable between the two molecules, and (ii) differently from E. coli produced rIL-21, S2 produced IL21-Ig-Fc was not immunogenic upon repeated doses. Indeed, None of the animals developed an anti-fusion protein antibody response (data not shown).

### Sample collection and processing

Collections and processing of blood, RB and LN were performed as previously described [26], [70], [71]. Briefly, blood samples have been used for a complete blood count and routine chemical analysis, and plasma separated by centrifugation within 1 h of phlebotomy. Peripheral blood mononuclear cells were prepared by density gradient centrifugation. For rectal biopsies, an anoscope has been placed a short distance into the rectum and up to 20 pinch biopsies obtained with biopsy forceps. RB-derived lymphocytes have been isolated by digestion with 1 mg/ml collagenase for 1 h at 37°C, and then passed through a 70-µm cell strainer to remove residual tissue fragments. For lymph node biopsies, the skin over the axillary or inguinal region have been clipped and surgically prepared. An incision has been made over the LN, which has been exposed by blunt dissection and excised over clamps. Biopsies have been homogenized and passed through a 70-µm cell strainer to mechanically isolate lymphocytes. All samples were processed, fixed (1% paraformaldehyde), and analyzed within 24 hours of collection.

### Flow cytometric analysis

Fourteen-parameter flow cytometric analysis was performed on whole blood, PBMC, LN and RB derived cells according to standard procedures using a panel of monoclonal antibodies that we and others have shown to be cross-reactive with RM [71], [72]. Predetermined optimal concentrations were used of the following antibodies: anti-CD3-Alexa700 (clone SP34-2), anti-CD3-APC-Cy7 (clone SP34-2), anti-CD4-PE (clone L200), anti-CD8-PacBlue (clone RPA-T8), anti-CD95-PE-Cy5 (clone DX2), anti-CCR5-APC (clone 3A9), anti-Ki-67-Alexa700 (clone B56), anti-CD62L-FITC (clone SK11), anti-IL-21-Alexa Fluor647 (clone 3A3-N2.1), anti-IFN-γ-PE-Cy7 (clone B27), anti-CD27-Alexa-700 (Clone M-T271), anti-CD21-PE-Cy5 (Clone B-ly4), (all from BD Pharmingen); anti β7-PE (clone FIB504), anti-IL-17-Alexa Fluor488 (clone eBio64DEC17), anti-CCR7-APC (clone 3D12) (all from eBioscience); anti-CD4-PacBlue (clone OKT4), anti-HLA-DR-APC-Cy7 (clone L243), anti-IL-2-Alexa700 (clone MQ1-17H12), anti-CD20-PerCPCy5.5 (clone 2H7) (all from Biolegend); anti-CD28-ECD (clone CD28.2) and anti-CD69-ECD (clone TP1.55.3) (Beckman Coulter); anti-CD8-Qdot705 (clone 3B5), anti-Granzyme B-ECD (Clone GB11) and Aqua Live/Dead amine dye-AmCyan (Invitrogen); anti-Perforin-FITC (clone Pf344, Mabtech); antiIgD FITC (catalog no. 2030-02, SouthernBiotech). A^*^01CM9-Gag_181–189_ tetramer-APC and PE antibodies were generously provided by Dr. David Watkins (Wisconsin National Primate Research Center, University of Wisconsin). Perforin and Granzyme B staining in total T cells was performed as described previously [57]. Flow cytometric acquisition was performed on at least 100,000 CD3^+^ T cells on an LSRII cytometer driven by the FACS DiVa software. Analysis of the acquired data was performed using FlowJo software (TreeStar).

### Intracellular cytokine staining

Levels of Th17 cells were determined as the percentage of CD4+ T cells that produce IL-17 following *in vitro* stimulation with PMA & Ionomycin. PBMC and RB derived cells, isolated as described above, were resuspended to 1×10^6^ cells/ml in complete RPMI 1640 medium. Cells were then incubated for 4 h at 37°C in medium containing PMA, A23187, and Golgi Stop. Following incubation, the cells were washed and stained with surface markers for 30 min in the dark at 4°C followed by fixation and permeabilization. After permeabilization, cells were washed and stained intracellularly with the antibody for the cytokines of interest for 1 h in the dark at 4°C. Following staining, cells were washed, fixed in PBS containing 1% paraformaldehyde, and acquired on an LSRII cytometer.

### Plasma levels of immune activation/inflammation markers and IL-22

LPS levels were measured in plasma (with EDTA as anticoagulant) by the limulus amebocyte lysate (LAL) chromogenic endpoint assay (Lonza Group Ltd, Allendale, NJ), as previously described [8]. Ten microliters of plasma was diluted 1∶10 in endotoxin-free water, and heat-inactivated at 85°C for 15 min to inactivate inhibitory plasma proteins. Results were calculated in relation to an *Escherichia coli* endotoxin standard provided with the assay, after background subtraction, and expressed in pg/mL. Plasma levels of sCD14 were quantified by using Human sCD14 Immunoassay kit (R&D systems, Minneapolis, MN). Ten microliters of plasma was diluted 400 fold by adding 3990 µl of calibrator diluent and assayed in duplicate as per manufacturers recommendations. Results of sCD14 were expressed in pg/ml. Levels of soluble tumor necrosis factor receptors II (sTNF-RII), CXCL10/IP10, D-Dimer and neopterine were measured in plasma using commercially available rhesus cross reacting human ELISA kits as per manufacturer's instructions. Levels of sTNFRII and IP-10 were quantified using human sTNFR II and IP-10 Quantikine ELISA kit (R&D systems, Minneapolis, MN) and expressed as pg/ml. D-Dimer was measured by IMUCLONE D-Dimer ELISA kit (American Diagnostica, Stamford, CT) and expressed as ng/ml. Neopterine levels in plasma were measured by a competitive ELISA (IBL International, Hamburg, Germany) and expressed as nmol/L. IL-22 levels were measured in plasma using monkey specific IL-22 ELISA kit (BlueGene, Life Sciences Advanced Technologies Inc, FL, USA), and expressed as pg/ml.

### Immunohistochemistry and quantitative image analysis

Immunohistochemistry was performed using a biotin-free polymer approach (Golden Bridge International, Inc.) on 5-µm tissue sections mounted on glass slides, which were dewaxed and rehydrated with double-distilled H_2_O. Heat induced epitope retrieval (HIER) was performed by heating sections in 10 mM Citrate (pH 6.0) in a pressure cooker set at 122°C for 30 s. After HIER, slides were rinsed in ddH_2_O and then loaded on an IntelliPATH autostainer (Biocare Medical) and stained with optimal conditions determined empirically that consisted of a blocking step using blocking buffer (TBS with 0.05% Tween-20 and 0.5% casein) for 10 min and an endogenous peroxidase block using 1.5% (v/v) H_2_O_2_ in TBS (pH 7.4) for 10 min. Rabbit polyclonal anti-myeloperoxidase (1∶1,000; Dako) was diluted in blocking buffer and incubated for 1 h at room temperature. Tissue sections were washed, and detected using the Rabbit Polink-1 HRP staining system (Golden Bridge International, Inc) according to manufacturer's recommendations. Sections were developed with Impact DAB (Vector Laboratories). All slides were washed in ddH_2_O, counterstained with hematoxylin, mounted in Permount (Fisher Scientific), and scanned at high magnification (×200) using the ScanScope CS System (Aperio Technologies) yielding high-resolution data from the entire tissue section. Representative regions of interest (ROIs; 500 mm2) were identified and high-resolution images extracted from these whole-tissue scans. The percent area of the lamina propria that stained for myeloperoxidase+ neutrophils were quantified using Photoshop CS5 and Fovea tools.

### Anti-SIV antibodies

Serum samples from IL-21-treated and control RMs were analyzed for their reactivity to viral antigens in a concanavalin A (ConA) ELISA [73]. Briefly, 96-well plates were coated with 5 µg of ConA for 1 h, washed and incubated with the SIV viral supernatant, which is first incubated with triton detergent. After blocking, sera at different dilution were added to the wells and incubated for 1 h at room temperature. The plates were washed and anti-SIV IgG Ab was detected after incubating 20 min with anti-human IgG biotin/streptavidin HRP.

### Statistical analysis

Repeated-measures analyses for each outcome (CD4^+^ T cells; CD4^+^Ki-67^+^ T cells; Th17 cells; CD8^+^Ki-67^+^; LPS; sCD14, etc) were performed with a means model with SAS Proc Mixed (version 9) providing separate estimates of the means by weeks post-infection and treatment group. A compound-symmetic variance-covariance form in repeated measurements was assumed for each outcome and robust estimates of the standard errors of parameters were used to perform statistical tests and construct 95% confidence intervals [74]. The model-based means are unbiased with unbalanced and missing data, so long as the missing data are non-informative (missing at random). T-tests were used to compare the differences between the model-based treatment means (least-squares means) at each time point within the framework of the mixed effects linear model. A Bonferroni adjustment (.05/5, P = 0.01) was used for the five treatment comparisons performed at wk −2, 2, 4, 6, and 23 post-infection. Statistical tests were 2-sided. Pearson product-moment correlation coefficients were utilized to estimate linear associations for normally distributed data (Figure 7B) and Spearman rank correlation coefficients (Figure 7A) were used for skewed and other non-normal distributions. A P value≤0.05 was considered statistically significant for the correlation analyses. The mean ± SEM were used as descriptive statistics for each continuous outcome.

## Supporting Information

Figure S1 Effects of IL-21 on intestinal T cell levels in SIV-infected RMs. Longitudinal assessment of intestinal CD4^+^ T cells expressed both as fraction of total CD3^+^ T cells (**A**) and as fold change relative to the percentages of CD4^+^ T cells at wk2 (**B**). Values are shown for individual IL-21-treated (depicted in orange) or control (depicted in black) RMs. Shaded area represents time of IL-21 treatment.
(TIFF)

Figure S2 Effects of IL-21 administration on perforin and GrB production by CD4^+^ T cells. Longitudinal assessment of the mean frequencies of (**A**) perforin and (**B**) GrB in gated live CD3^+^CD4^+^ T cells from PBMC (left panels), LN (middle panels) and RB (right panels). IL-21-treated animals are depicted in orange, controls in black. Shaded area represents time of IL-21 treatment. Averaged data are presented as mean ± SEM.
(TIFF)

Figure S3 Effects of IL-21 administration on the frequency of B cell subsets and on anti-SIV antibodies. Circulating B cell subsets were analyzed longitudinally by flow cytometry. (**A**) Mean frequencies of memory B cells (CD3^-^CD20^+^CD21^hi^CD27^+^) and (**B**) swich memory B cells (CD3^−^CD20^+^CD21^hi^CD27^+^IgD^−^) in control and IL-21-treated animals. (**C**) Longitudinal assessment of plasma levels of anti-SIV antibodies in the two groups of animals. IL-21-treated RMs are depicted in orange, controls in black. Shaded area represents time of IL-21 treatment. Averaged data are presented as mean ± SEM.
(TIFF)

Figure S4 Effects of IL-21 on the frequency of blood and intestinal CD4^+^ T cells expressing IL-17, IFN-γ and IL-2 in SIV-infected RMs. Longitudinal assessment of the percentages of circulating (**A–C**) or intestinal (**D–F**) CD4^+^ T cells that express IL-17 (**A, D**), IFN-γ (**B, E**) or IL-2 (**C, F**) in IL-21-treated (orange) and control (black) RMs. Shaded area represents time of IL-21 treatment. Averaged data are presented as mean ± SEM.
(TIFF)

Figure S5 Effects of IL-21 on plasma levels of IL-22 in SIV-infected RMs. Plasma levels of IL-22 (pg/ml) were determined in IL-21-treated (orange) and control (black) RMs. Data are shown as fold change variation at wk6 (end of treatment) and wk23 (end of study) as compared to wk2 (pre-treatment) p.i. Averaged data are presented as mean ± SEM.
(TIFF)

Figure S6 Effects of IL-21 on intestinal T cell proliferation and microbial translocation in SIV-infected RMs. (**A, B**) Longitudinal assessment of intestinal (**A**) CD4^+^Ki-67^+^ and (**B**) CD8^+^Ki-67^+^ T cells in IL-21-treated and control RMs. (**C, D**) Longitudinal assessment of plasma levels of (**C**) LPS and (**D**) sCD14 in IL-21-treated and control RMs. Values are shown for individual IL-21-treated (depicted in orange) or control (depicted in black) RMs. Shaded area represents time of IL-21 treatment.
(TIFF)

Figure S7 Effects of IL-21 on systemic T cell activation and proliferation in SIV-infected RMs. Longitudinal assessment of the percentage of circulating (**A**) CD4^+^Ki-67^+^, (**B**) CD8^+^Ki-67^+^, (**C**) CD4^+^PD-1^+^, and (**D**) CD8^+^PD-1^+^ T cells in IL-21-treated (orange) and control (black) RMs. Shaded area represents time of IL-21 treatment. Averaged data are presented as mean ± SEM.
(TIFF)

Table S1 Modifications induced by IL-21 treatment on several immunological parameters. Summary of the parameters that were significantly different between IL-21-treated and control animals in at least one experimental time point. NA: not analyzed; NS: Not significant.
(DOCX)
