# Supplementary figures and images for: Maintenance of Intestinal Th17 Cells and Reduced Microbial Translocation in SIV-infected Rhesus Macaques Treated with Interleukin (IL)-21
Source: PLoS Pathog. 2013 Jul 4;9(7):e1003471. doi: 10.1371/journal.ppat.1003471 (PMC3701718; doi:10.1371/journal.ppat.1003471)

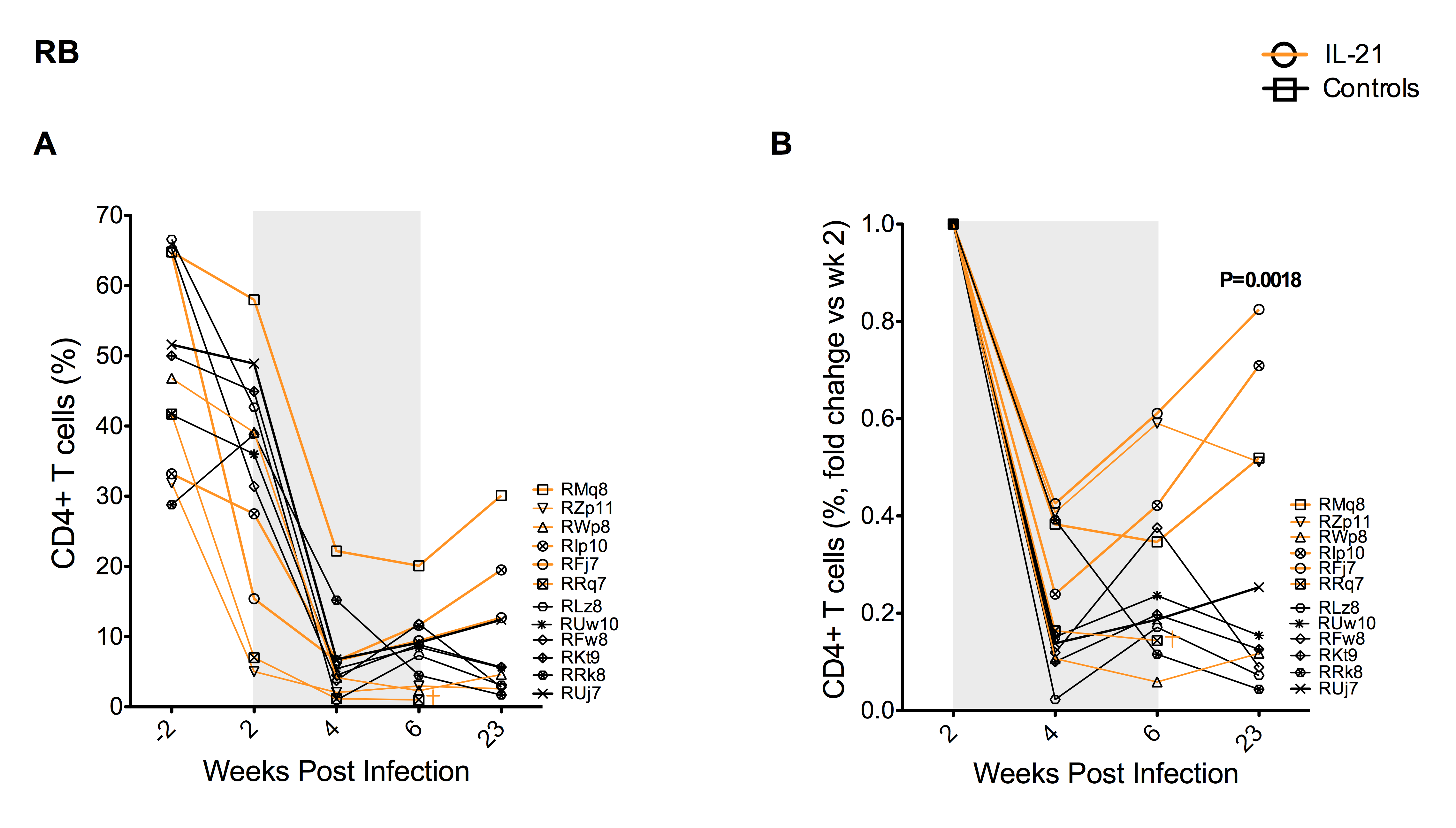

Supplement: Figure S1 — Effects of IL-21 on intestinal T cell levels in SIV-infected RMs. Longitudinal assessment of intestinal CD4+ T cells expressed both as fraction of total CD3+ T cells (A) and as fold change relative to the percentages of CD4+ T cells at wk2 (B). Values are shown for individual IL-21-treated (depicted in orange) or control (depicted in black) RMs. Shaded area represents time of IL-21 treatment. (TIFF) [file ppat.1003471.s001.tiff]

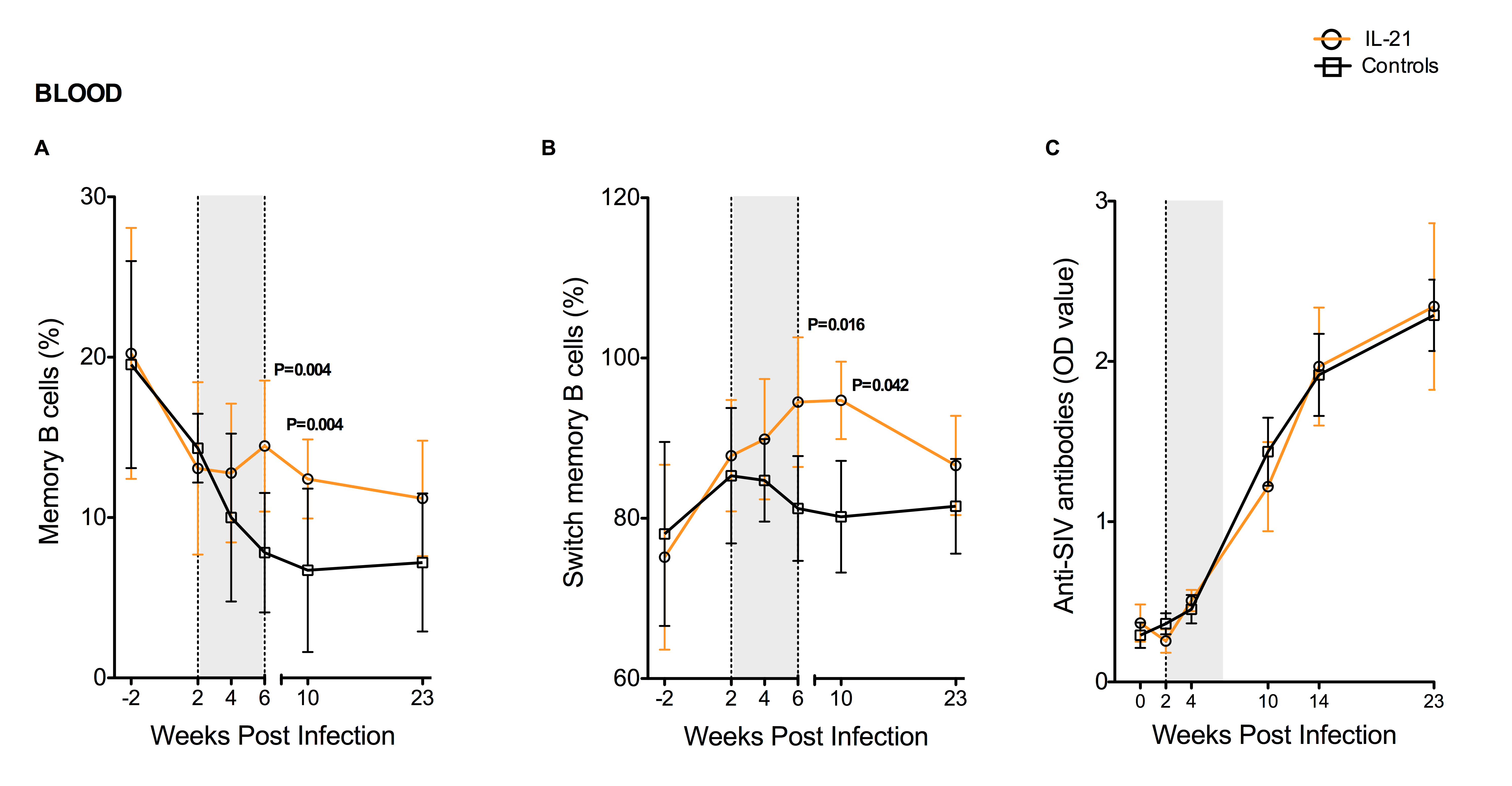

Supplement: Figure S3 — Effects of IL-21 administration on the frequency of B cell subsets and on anti-SIV antibodies. Circulating B cell subsets were analyzed longitudinally by flow cytometry. (A) Mean frequencies of memory B cells (CD3-CD20+CD21hiCD27+) and (B) swich memory B cells (CD3−CD20+CD21hiCD27+IgD−) in control and IL-21-treated animals. (C) Longitudinal assessment of plasma levels of anti-SIV antibodies in the two groups of animals. IL-21-treated RMs are depicted in orange, controls in black. Shaded area represents time of IL-21 treatment. Averaged data are presented as mean ± SEM. (TIFF) [file ppat.1003471.s003.tiff]

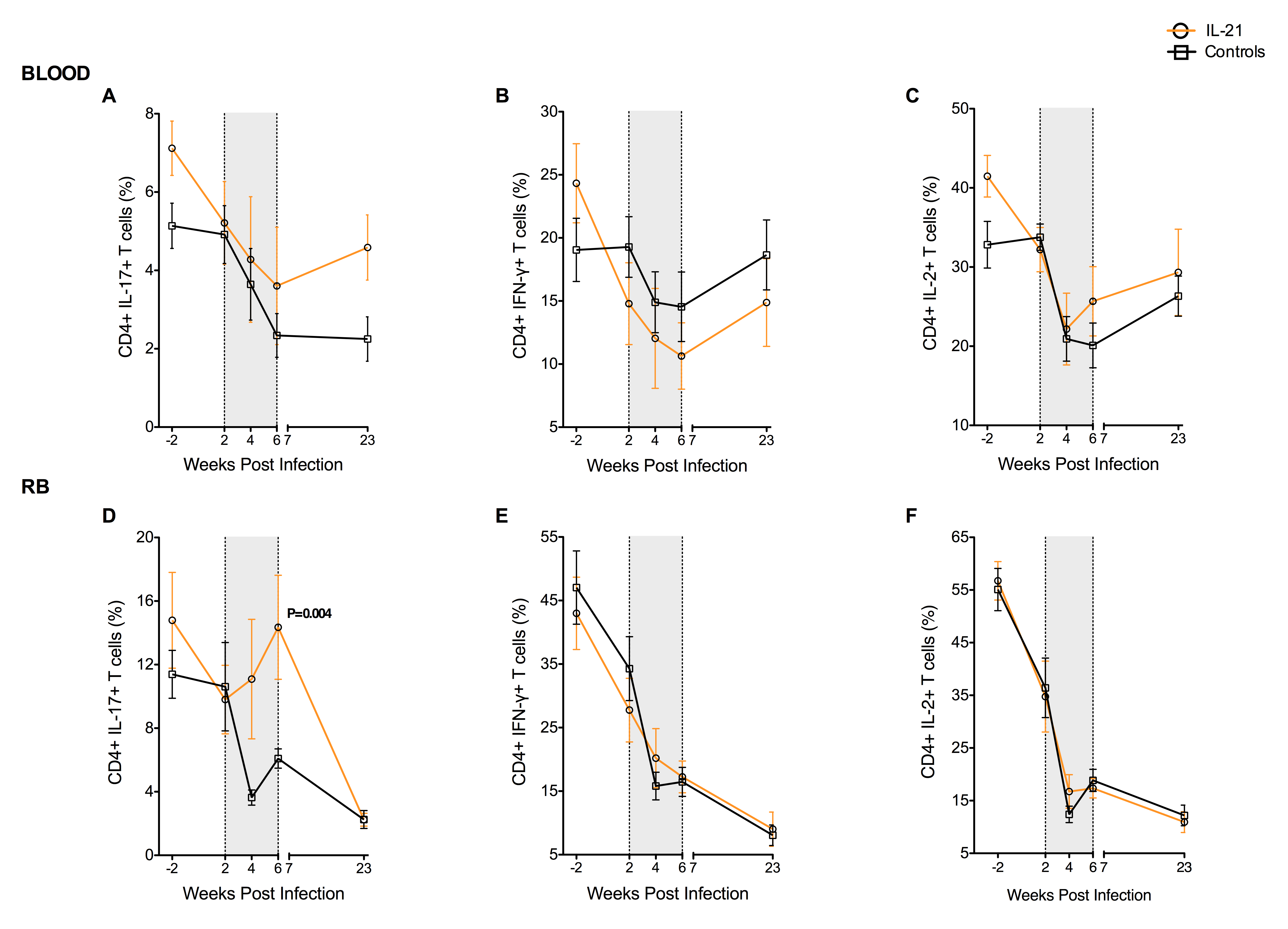

Supplement: Figure S4 — Effects of IL-21 on the frequency of blood and intestinal CD4+ T cells expressing IL-17, IFN-γ and IL-2 in SIV-infected RMs. Longitudinal assessment of the percentages of circulating (A–C) or intestinal (D–F) CD4+ T cells that express IL-17 (A, D), IFN-γ (B, E) or IL-2 (C, F) in IL-21-treated (orange) and control (black) RMs. Shaded area represents time of IL-21 treatment. Averaged data are presented as mean ± SEM. (TIFF) [file ppat.1003471.s004.tiff]

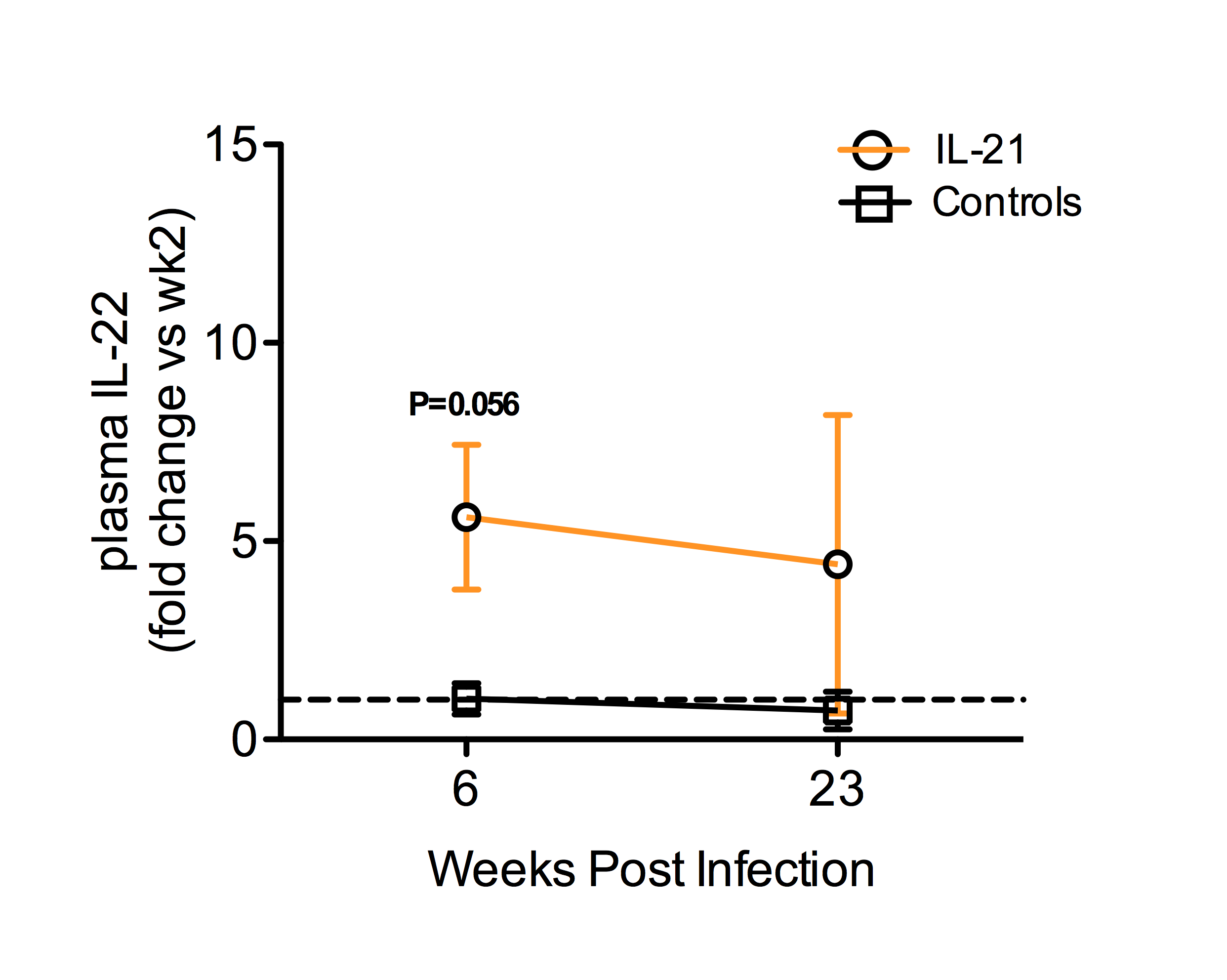

Supplement: Figure S5 — Effects of IL-21 on plasma levels of IL-22 in SIV-infected RMs. Plasma levels of IL-22 (pg/ml) were determined in IL-21-treated (orange) and control (black) RMs. Data are shown as fold change variation at wk6 (end of treatment) and wk23 (end of study) as compared to wk2 (pre-treatment) p.i. Averaged data are presented as mean ± SEM. (TIFF) [file ppat.1003471.s005.tiff]

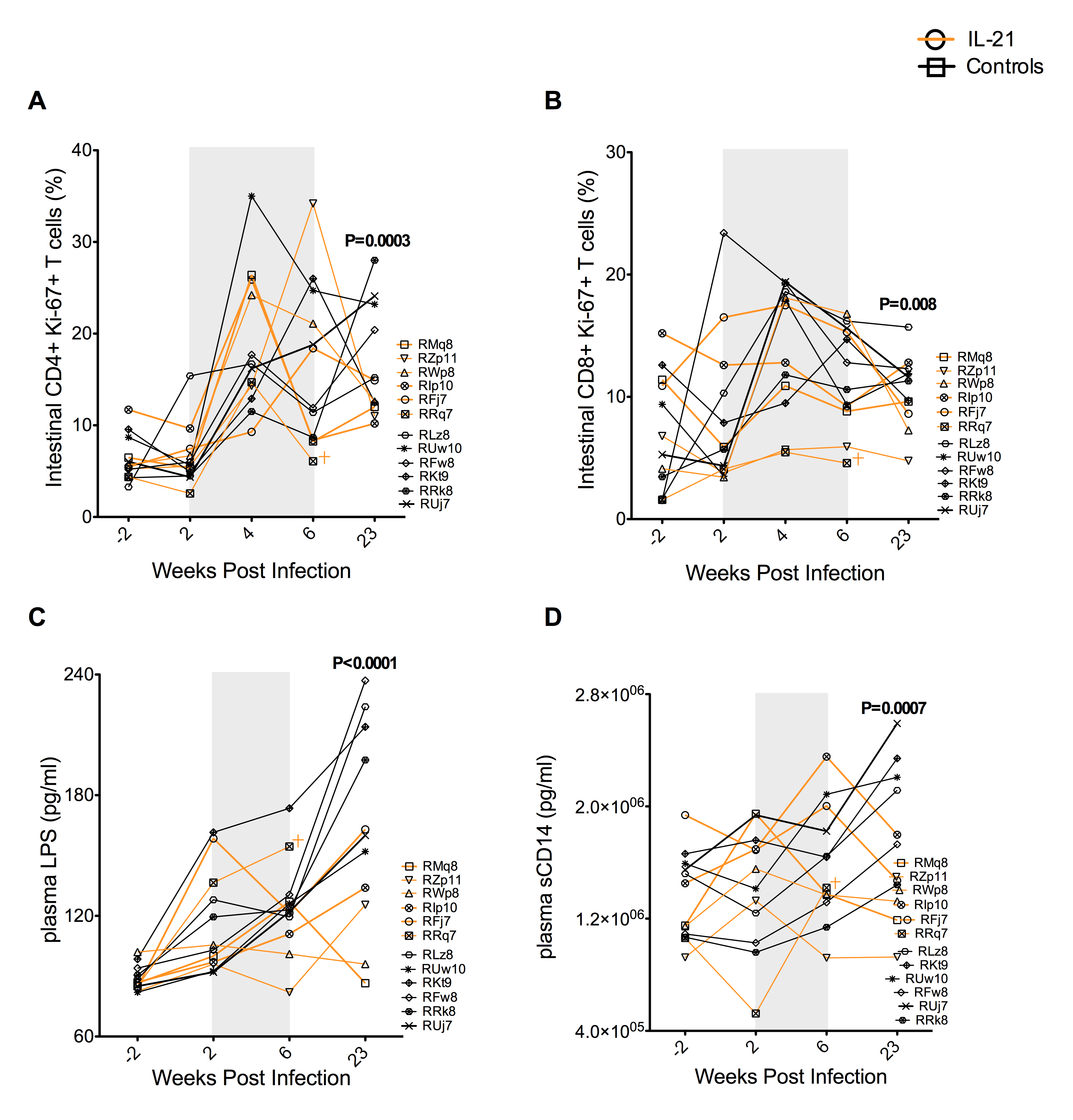

Supplement: Figure S6 — Effects of IL-21 on intestinal T cell proliferation and microbial translocation in SIV-infected RMs. (A, B) Longitudinal assessment of intestinal (A) CD4+Ki-67+ and (B) CD8+Ki-67+ T cells in IL-21-treated and control RMs. (C, D) Longitudinal assessment of plasma levels of (C) LPS and (D) sCD14 in IL-21-treated and control RMs. Values are shown for individual IL-21-treated (depicted in orange) or control (depicted in black) RMs. Shaded area represents time of IL-21 treatment. (TIFF) [file ppat.1003471.s006.tiff]

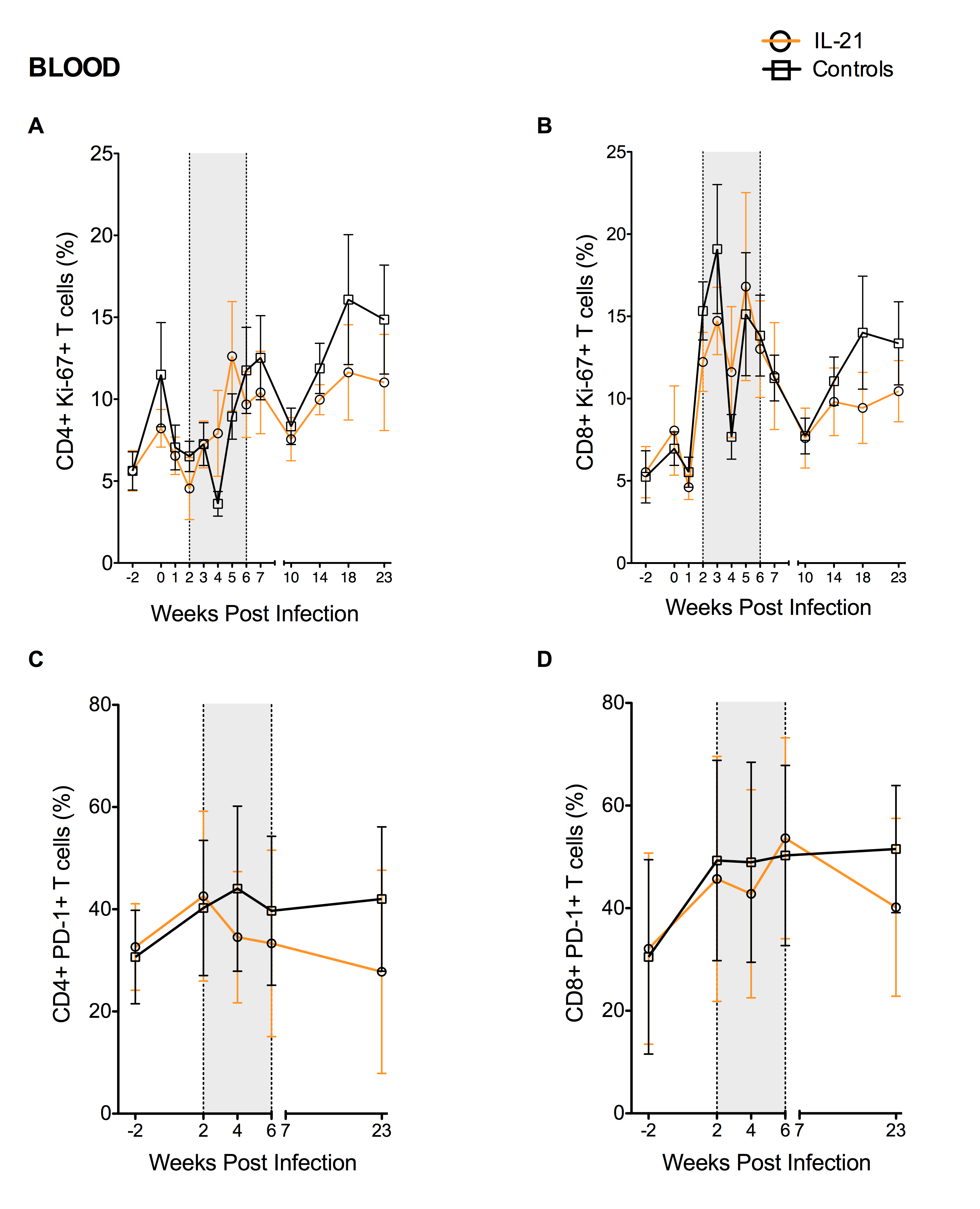

Supplement: Figure S7 — Effects of IL-21 on systemic T cell activation and proliferation in SIV-infected RMs. Longitudinal assessment of the percentage of circulating (A) CD4+Ki-67+, (B) CD8+Ki-67+, (C) CD4+PD-1+, and (D) CD8+PD-1+ T cells in IL-21-treated (orange) and control (black) RMs. Shaded area represents time of IL-21 treatment. Averaged data are presented as mean ± SEM. (TIFF) [file ppat.1003471.s007.tiff]
