# Supplementary material for: Maintenance of Intestinal Th17 Cells and Reduced Microbial Translocation in SIV-infected Rhesus Macaques Treated with Interleukin (IL)-21
Source: PLoS Pathog. 2013 Jul 4;9(7):e1003471. doi: 10.1371/journal.ppat.1003471 (PMC3701718; doi:10.1371/journal.ppat.1003471)
Supplement: Table S1 — Modifications induced by IL-21 treatment on several immunological parameters. Summary of the parameters that were significantly different between IL-21-treated and control animals in at least one experimental time point. NA: not analyzed; NS: Not significant. (DOCX) [file ppat.1003471.s008.docx]

**Supplementary table 1**

Summary of the parameters that were significantly different between IL-21-treated and control animals in at least one experimental time point. NA: not analyzed; NS: Not significant.

| **Parameters** | **Blood** | **RB** | **Effect on**  **IL-21 treated RMs** |
| --- | --- | --- | --- |
| CD4 (%, fold change over wk2) | NS | wk23 | Increase |
| CD8+Perf+ (%) | Wk6, Wk10 | Wk6 | Increase |
| CD8+GrB+ (%) | Wk6, Wk10 | Wk6 | Increase |
| CD8+Tet+Perf+ (%) | Wk6, Wk10 | Wk4, Wk6 | Increase |
| CD8+Tet+GrB+ (%) | Wk6, Wk10 | Wk6 | Increase |
| CD4+IL-17+ (%) | NS | Wk6 | Increase |
| CD4+Ki67+ (%) | NS | Wk23 | Decrease |
| CD8+Ki67+ (%) | NS | Wk23 | Decrease |
| Memory B cells (%) | Wk6, Wk10 | NA | Increase |
| Switch memory B cells (%) | Wk6, Wk10 | NA | Increase |
| LPS (pg/ml) | Wk23 | NA | Decrease |
| sCD14 (pg/ml) | Wk23 | NA | Decrease |
| sTNF-RII (fold change over wk2) | Wk6, Wk23 | NA | Decrease |
| IP-10 (fold change over wk2) | Wk23 | NA | Decrease |
| Neopterine (fold change over wk2) | Wk23 | NA | Decrease |
| IL-22 (fold change over wk2) | Wk6 (0.056) | NA | Increase |
